# Autologous adipose-derived mesenchymal stem cell therapy reverses detrusor underactivity: open clinical trial

**DOI:** 10.1186/s13287-023-03294-8

**Published:** 2023-04-05

**Authors:** Henrique Rodrigues Scherer Coelho, Silvia Cordeiro das Neves, Jovino Nogueira da Silva Menezes, Andréia Conceição Milan Brochado Antoniolli-Silva, Rodrigo Juliano Oliveira

**Affiliations:** 1 Centro de Estudos em Células-Tronco, Terapia Celular e Genética Toxicológica (CeTroGen), Faculdade de Medicina (FAMED), Universidade Federal do Mato Grosso do Sul (UFMS), Campo Grande, Mato Grosso do Sul Brazil; 2 Programa de Pós-graduação em Saúde e Desenvolvimento na Região Centro-Oeste, Faculdade de Medicina (FAMED), Universidade Federal do Mato Grosso do Sul (UFMS), Campo Grande, Mato Grosso do Sul Brazil; 3 Clinica Samari, Campo Grande, Mato Grosso do Sul Brasil

**Keywords:** Cell therapy, Urinary bladder, Urodynamics

## Abstract

**Background:**

Detrusor underactivity is a disease that can cause chronic urinary tract infection, urinary tract infection, urinary retention and kidney failure and has no effective treatment in traditional medicine. The present research evaluated the effects of cell therapy with adipose tissue-derived stem cells on the treatment of detrusor underactivity in men.

**Methods:**

Nine male patients diagnosed with a clinical and urodynamic diagnosis of detrusor underactivity were evaluated and underwent two transplants via cystourethroscopy, with 2 × 10^6^ cells/transplant, performed by intravesical injection at five points on the bladder body above the vesical trigone.

**Results:**

Cell therapy increased the maximum flow from 7.22 ± 1.58 to 13.56 ± 1.17, increased the mean flow from 3.44 ± 0.74 to 5.89 ± 0.45, increased the urinated volume from 183.67 ± 49.28 to 304.78 ± 40.42 and reduced the residual volume in the uroflowmetry exam from 420.00 ± 191.41 to 118.33 ± 85.51; all of these changes were significant (*p* < 0.05). There were also significant increases (*p* < 0.05) in maximum flow (from 7.78 ± 0.76 to 11.56 ± 1.67), maximum detrusor pressure (from 20.22 ± 8.29 to 41.56 ± 5.75), urinary volume (from 244 ± 27.6 to 418.89 ± 32.73) and bladder contractility index (from 44.33 ± 4.85 to 100.56 ± 8.89) in the pressure flow study. Scores on the International Consultation on Incontinence Questionnaire decreased from 11.44 ± 1.43 to 3.78 ± 0.78 after cell therapy, which indicates an improvement in quality of life and a return to daily activities. No complications were observed in the 6-month follow-up after cell therapy. Before treatment, all patients performed approximately five intermittent clean catheterizations daily. After cell therapy, 7/9 patients (77.78%) did not need catheterizations, and the number of catheterizations for 2/9 patients (22.28%) was reduced to two catheterizations/day.

**Conclusions:**

The results indicate that stem cell therapy led to improvements in voiding function. Cell therapy with adipose tissue-derived stem cells is safe and should be considered a new therapeutic option for the treatment of detrusor underactivity.

*Trial registration* ISRCTN, ISRCTN23909398; Registered 15 March 2021—Retrospectively registered, https://doi.org/10.1186/ISRCTN23909398

**Supplementary Information:**

The online version contains supplementary material available at 10.1186/s13287-023-03294-8.

## Background

Underactive bladder is characterized by slow urinary flow, hesitancy straining to urinate, with or without a feeling of incomplete bladder emptying and elevated postvoid residual volume [1]. Detrusor underactivity (DU) is characterized by low pressure or short detrusor contraction associated with low urinary flow [2].

The incidence of voiding dysfunction in children can be as high as 1/5 [3]. Among adults under 50 years of age, voiding dysfunction affects up to 28% of the population, and among individuals over 70 years of age, this figure rises to 48% [4].

To date, no traditional medicine has been shown to treat DU and restore bladder contractility. Thus, patients affected by this disease often experience an increase in the severity of the signs and symptoms.

The main factors complicating treatment are high postvoid residual volumes, recurrent urinary infections, formation of urinary tract stones and even acute insufficiency that can progress to chronic kidney disease [4–7]. Therefore, it is necessary to develop new therapies, as clean intermittent catheterization physical therapy and clinical follow-up can only be used to treat the complications of the disease but do not restore the contractile capacity of the bladder.

Cell therapy (CT) with mesenchymal stem cells (MSCs) is a potential treatment. A previous study reported the autologous use of muscle-derived stem cells to treat bladder DU: the results demonstrated that the therapy is safe and reduces the maximum cystometric capacity (MCC), but the patient remained dependent on self-catheterization [8].

As CT is still relatively unexplored in this area, the present research is the first to examine the evaluation of the effects of CT with adipose tissue-derived stem cells (ADSCs) in the treatment of DU among men.

## Patients and methods

### Trial design

The current open clinical trial was carried out at the Maria Aparecida Pedrossian University Hospital (HUMAP) with approval from the National Research Ethics Committee (CONEP) and the Human Research Ethics Committee (CEP) of the Federal University of Mato Grosso do Sul (CEP/CONEP) under No. 2,745,746. The study was carried out in accordance with Resolution No. 466 of the National Health Council (Brasil, 2012) and registered in ISRCTN 23909398 and in the Brazilian Registry of Clinical Trials—REBEC—RBR-10bk2qbb.

### Participants

Nine male patients from the Urology Outpatient Clinic of HUMAP with a clinical and urodynamic diagnosis of DU were evaluated between November 2018 and July 2021. The individuals were informed about the purpose of the research, and those who agreed to participate signed the Free and Informed Consent Form (FICF).

The inclusion criteria were male patients with a urodynamic study showing only DU without the presence of an obstructive factor to urinary flow, patients still undergoing intermittent clean catheterization, and patients who have not undergone any surgical procedure to the lower urinary tract in the last 12 months. The exclusion criteria were end-stage renal failure (with oligoanuria), recurrent urinary infection, nonadherence to clinical follow-up protocols, and nonadherence to intermittent clean self-catheterization. Additional exclusion criteria were the presence of a recently treated malignant neoplasm, malignancy confirmed in treatment, or suspected cancer, as these criteria were contraindications to the use of stem cells.

### Intervention

The patients who were followed up on an outpatient basis underwent periodic clinical and laboratory evaluations, including presurgical examinations. For diagnosis, patients also underwent urinary tract and prostate ultrasound, cystourethroscopy (30° lens, 19 fr cystoscope, coupled to a Storz camera) and urodynamic study consisting of initial uroflowmetry, differential cystometry and a pressure flow study performed with urodynamic equipment (Dynapack MPX 816—Poligrafo Dynamed/São Paulo-SP, Anvisa registration 80021460001).

### Urodinamic study

The urodynamic study was divided into three phases: uroflowmetry, cystometry and flow-pressure study.

Uroflowmetry is the first phase of the exam and is also called free urinary fluid. It allows us to obtain the maximum flow, mean flow and urination volume. Additionally, it provides postvoiding residue that can be measured by ultrasound or vesical catheterization.

Cytometry is the second phase of the exam and is performed with vesical catheterization with two urethral tubes, an 8-French probe for bladder filling with 0.9% saline solution and a 6-French urethral probe to measure intravesical pressure during bladder filling and emptying. A catheter with a cuff was inserted into the rectal ampulla to measure intra-abdominal pressure. During bladder filling, the following data were obtained: maximum cystometric capacity, compliance, presence of urinary leakage with effort maneuvers and detrusor contraction.

The flow-pressure assessment was the last phase of the urodynamic study. The patient was asked to start voiding with the urethral tubes still in place to assess detrusor pressure and maximal flow during voiding. Thus, it is possible to assess bladder emptying and conclude whether there is normal or reduced detrusor contraction [9].

Patients diagnosed with DU who agreed to participate in the research and signed the free and informed consent form were immediately included in the study, and the tests performed during the diagnosis were used as time zero (start of the study). At that moment, the patients also answered the International Consultation on Incontinence Questionnaire-Short-Form (ICIQ-SF). T0 was considered the control situation, that is, how the patients were before any intervention. A new evaluation was performed 60 days after the end of cell therapy (T1). Twelve months (T3) after the end of cell therapy, the patients answered the ICIQ-SF questionnaire again. The ICIQ-SF is a validated and widely used international questionnaire in the area [10, 11].

The adipose tissue for the extraction of MSCs was collected from the inner face of the right and left thighs through liposuction of each patient. The procedures were performed by a specialist in plastic surgery on an outpatient basis. For collection, aseptic technique was performed with 4% aqueous chlorhexidine, and sterile drapes were placed. Anesthesia was then administered with 125 mL of an anesthetic solution (20% lidocaine without adrenaline, 0.9% saline and 8.4% sodium bicarbonate) to promote tissue lipodistention. Then, with the aid of a liposuction cannula (3 mm), 200 mL of solution was removed. The material was placed in a sterile flask containing phosphate buffer solution (PBS) with 1% antibiotic/antimycotic (streptomycin/penicillin/amphotericin B, Sigma®, Lot 097M4875V). The material was transported to the Center for Studies in Stem Cells, Cell Therapy and Toxicological Genetics (CeTroGen). The extraction, cultivation, expansion and characterization of cells were performed according to Pesarini [12] with adaptations (Additional file 1).

After 60 days, the cells were trypsinized, washed to exhaustion with PBS and transplanted by means of cystourethroscopy via the urethra in an outpatient setting under local anesthesia. Twenty grams of 2% lidocaine gel was used.

Each patient received two transplants with 2 × 10^6^ ADSCs 30 days apart. Transplantation was performed by intravesical injection with a cystoscopic needle (20 gauge) at five points on the bladder body above the vesical trigone.

Patients were clinically followed up during and after transplantation to check for possible complications, including laboratory tests (Additional file 2).

### Data analysis

Data are presented as the mean ± standard deviation and/or percentages.

Times T0, T1 and T2 were compared using GraphPad Prism software (version 3.02; Graph-Pad Software Inc., San Diego, CA, USA). To compare T0 and T1, Student’s t-test was used, and to compare T0, T1 and T2, ANOVA/Tukey’s test was used. Differences were considered statistically significant when *p* < 0.05.

Data from the uroflowmetry study were used to describe the effectiveness of the treatment. The analyzed data included voided volume, residue, and voiding efficiency before and after CT treatment. The calculation was performed using the following formula: Bladder emptying efficiency = (Urinated volume/(Urinated volume + Residual)) × 100% [13].

## Results

### Demographics, comorbidities and adverse effects

Patient ages ranged from 53 to 74 years, with a mean age of 66 years. The etiology included 3 patients (33.3%) with benign prostatic hyperplasia, 2 patients (22.2%) with diabetes mellitus, 1 patient (11.1%) with urethral stricture, and 3 idiopathic patients (33.3%). Regarding comorbidities, 2 patients (22.2%) had diabetes mellitus, and 7 patients (77.7%) had systemic arterial hypertension. All patients (100%) required intermittent clean catheterization for bladder emptying (Table 1). No serious adverse effects related to ADSCs transplantation were observed. Adverse events related to fat collection and intravesical injection are listed in Table 2. The adverse events observed for the intravesical injection were already observed in other procedures performed by the group that did not involve the transplantation of ADSCs. For example, the administration of botulinum toxin is mentioned (data not shown).

**Table 1:** Patient demographics

	Total
Patients	9
Mean age ± SEM	65.66 ± 2.49
Etiology	
Benign prostatic hyperplasia	3/9 (33.3%)
Diabetes mellitus	2/9 (22.2%)
Urethral stricture	1/9 (11.1%)
Idiopathic	3/9 (33.3%)
Comorbidities	
Diabetes mellitus	2/9 (22.2%)
Systemic arterial hypertension	7/9 (77.7%)
Absent	2/9 (22.2%)
Bladder emptying	
Intermittent clean catheterization	9/9 (100%)

**Table 2:** Adverse effects

Procedure related event	Number of patients
Related to fat collection	
Ecchymosis	4
Edema	1
Pain	3
Related to intravesical injection	
Urinary infection	2
Hematuria	1
Dysuria	1

### Uroflowmetry

**Fig. 1:**
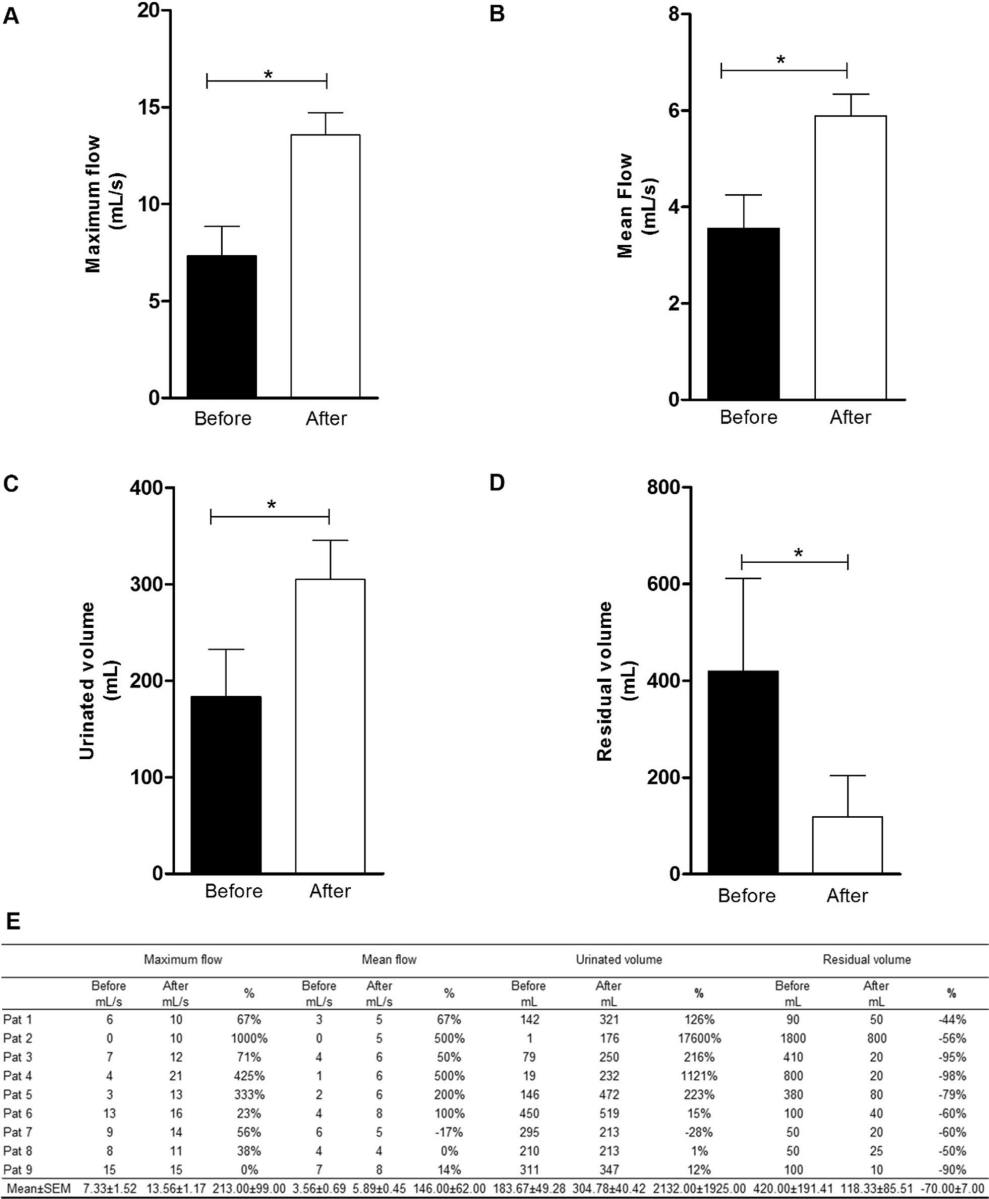
Mean Values ± Mean Standard Error obtained from the uroflowmetry exam of patients before and 60 days after ADSCs transplantation. **A** Maximum flow; **B** Mean flow; **C** Urinated volume; **D** Residual volume; **E** Maximum flow before and after the intervention, percentage change in maximum urinated flow, mean flow before and after the intervention, percentage change in mean urinated flow, absolute urinated volume before and after the intervention, percentage change in urinated volume, absolute residual volume before and after the intervention, percentage change in residual volume. Pat - Patient; % - percentage variation (Statistics: t-Student Peer, *p* < 0.05)

In the uroflowmetry exam, an increase (*p* < 0.05) in the maximum flow and in the mean flow of patients was observed 60 days after the last transplant. The initial mean maximum flow was 7.22 ± 1.58 mL/s, and after therapy, it increased to 13.56 ± 1.17 mL/s (Fig. 1A). The initial mean flow was 3.44 ± 0.74 mL/s before therapy and increased to 5.89 ± 0.45 mL/s after transplantation (Fig. 1B). According to Reynard [14], the normal maximum flow is above 15 mL/s, and patients with a maximum flow below 10 mL/s have voiding dysfunction (urinary jet alteration). A more specific analysis (patient by patient) showed that before therapy, patients 1–5, 7 and 8 had a peak flow < 10 mL/s, while patients 6 and 9 were borderline for voiding dysfunction. After CT, patients 4, 6 and 9 reached normal values (> 15 mL/s), while patients 1–3, 5, 7 and 8 had increased peak flow. The percentage variation in the maximum flow increase was between 0 and 900% (Fig. 1E). The voided volume increased after therapy (*p* < 0.05). The initial mean urinated volume was 183.67 ± 49.28 mL and changed to 304.78 ± 40.42 mL after the intervention (Fig. 1C). All patients except patient 7 had increased urination volume after CT. The highest percentage of increase in urinated volume was 17,600% in patient 2. Patient 7, the only patient who did not present an increase, actually presented a 28% reduction in urinated volume (Fig. 1E).

All patients had a reduction (*p* < 0.05) in residual urine volume. The mean residual volume was 420.00 ± 191.41 mL before CT and 118.33 ± 85.51 mL after CT (Fig. 1D). The lowest percentage of residual volume reduction was 44%, and the highest was 98% (Fig. 1E). According to D'Ancona [2], the residual volume must be < 50 mL. In a more detailed analysis, it was observed that initially, only patients 7 and 8 had a normal residual volume. All other patients had a residual volume > 50 mL. After CT, only patients 2 and 5 did not reach normal parameters. However, patient 2 had a residual volume reduction of 56%, and patient 5 had a residual volume reduction of 79% (Fig. 1E).

**Fig. 2:**
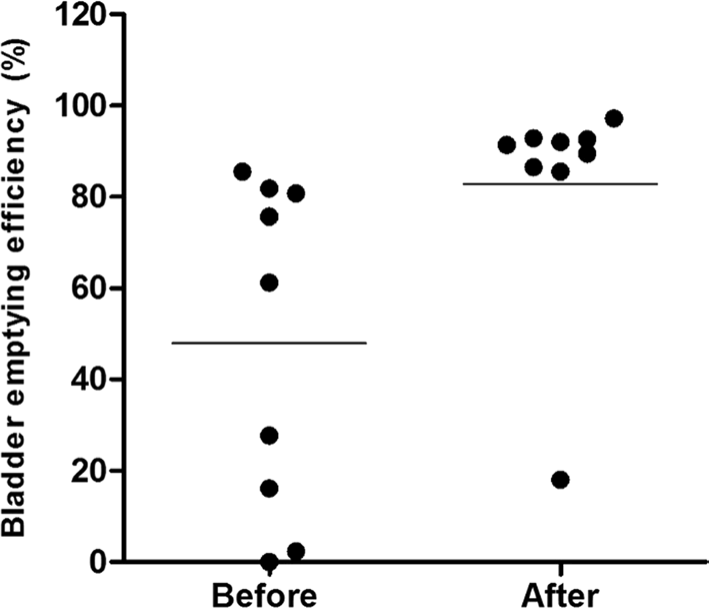
Percentage of bladder emptying efficiency before and after CT intervention

The bladder emptying efficiency improved in all patients after treatment with CT, with eight patients showing a percentage above 85% (Fig. 2).

### Cystometry

**Fig. 3:**
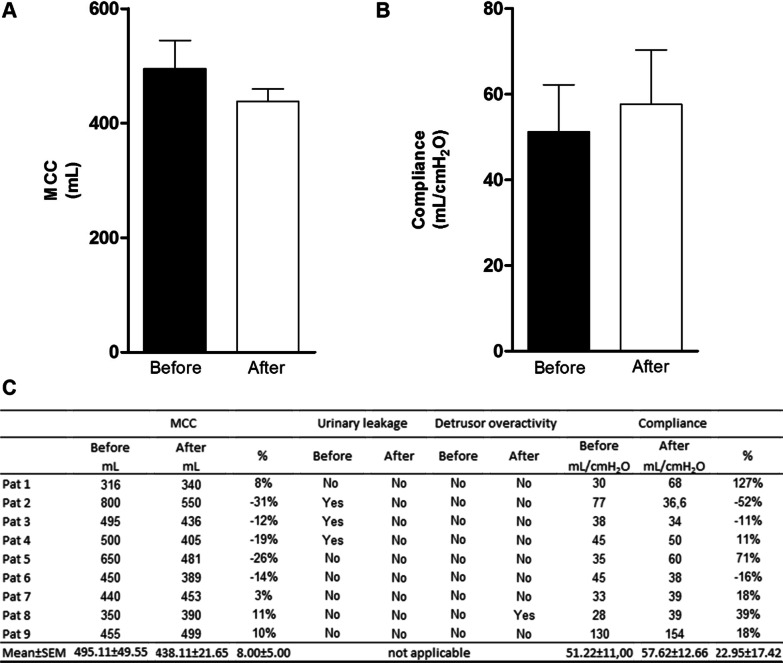
Mean values ± standard error of the mean maximum cystometric capacity, urinary leakage, detrusor overactivity and compliance obtained from the cystometry exam of patients before and 60 days after ADSCs transplantation. Pat – Patient; MCC – maximum cystometric capacity before and after the intervention; % – percentage change (Statistics: Paired t-Student, *p* < 0.05)

The MCC showed no significant differences before and after CT (Fig. 3A). In the cystometric examination, it was found that the MCC increased for patients 1 and patients 7–9 (44.45%) and decreased for patients 2–6 (55.55%). The percentage change in MCC ranged from -31% to 11%. According to D'Ancona [2], the MCC should be between 350 and 500 mL. Initially, patient 1 had a lower value than the reference, while patients 2 and 5 had higher values. After CT, patient 1 had an increased MCC that did not fall within the normal range. Patient 2 had a reduced MCC that was also not within the normal range. Patient 5 had a decreased MCC that was within the reference values (Fig. 3C).

Urinary leakage was frequent in 33.33% of patients before CT. After therapy, no patients had urinary leakage. Only patient 8 (11.11%) had uninhibited detrusor contraction during the assessment (Fig. 3C). Mean compliance did not differ (*p* = 0.05) before and after CT (Fig. 3B). This parameter increased in patients 1, 4, 5, 7, 8, and 9; the minimum percentage change was 11%, and the highest was 127%. Patients 2, 3 and 6 had reduced compliance; the smallest percentage change was 11%, and the largest was 52% (Fig. 3C).

### Pressure flow study

**Fig. 4:**
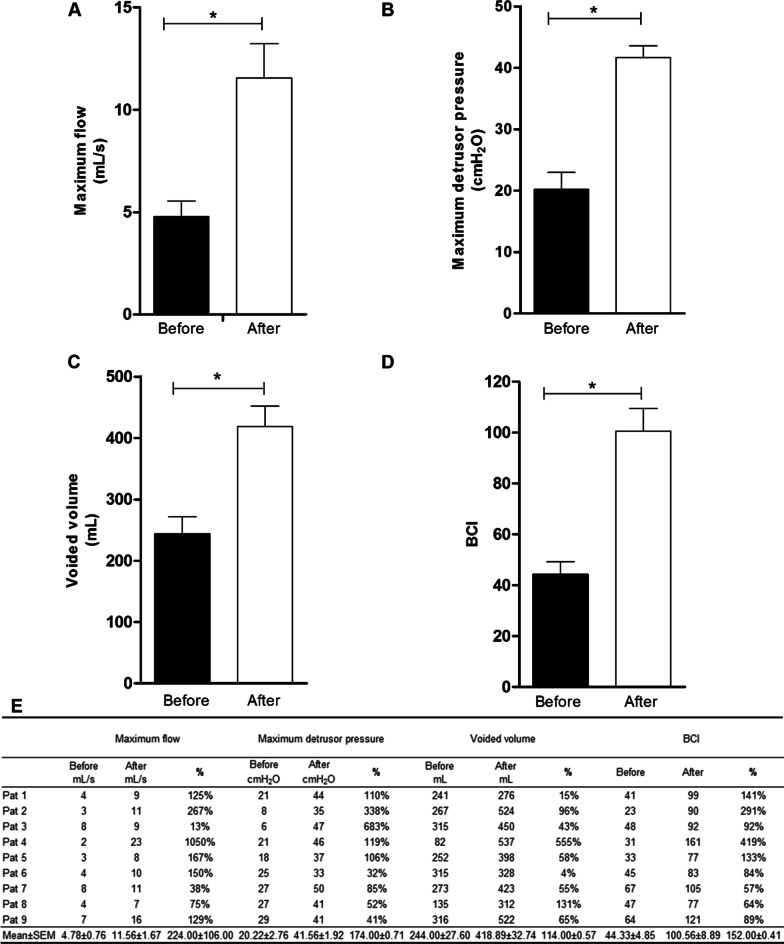
Mean Values ± Mean Standard Error obtained in the Pressure flow study of patients before and 60 days after ADSCs transplantation. **A** Maximum flow; **B** Maximum detrusor pressure; **C** Voided volume; **D** Bladder Contractility Index (BCI); **E** Maximum flow, percentage change in urination volume increase, Maximum detrusor pressure, percentage change in urination volume increase, Voided volume, percentage change in urination volume increase, Bladder Contractility Index (BCI). Pat – Patient; % - Percentage change; BCI – Bladder Contractility Index (Statistics: Paired t-Student, p < 0.05)

The pressure flow test showed that the maximum flow increased (*p* < 0.05) after ADSCs therapy. The initial mean peak flow was 4.78 ± 0.76 mL/s before CT. After transplantation, the maximum flow increased to 11.56 ± 1.67 mL/s (Fig. 4A). According to Ahmed [15], the maximum flow in the pressure flow assessment should be > 15 mL/s. Initially, all patients had a maximum flow lower than the recommended value. After CT, only patients 4 and 9 exhibited normal parameters. However, the maximum flow of the other patients improved by 13 to 267% (Fig. 4E).

The mean maximum detrusor pressure increased (*p* < 0.05) after CT (Fig. 4B). The initial mean value of the maximum detrusor pressure was 20.22 ± 8.29 cm/H_2_O and changed to 41.56 ± 5.75 cm/H_2_O. The percentage increase variation ranged from 32 to 683%. Zerati Son, Nardoza Junior, and Reis [16] recommend that the maximum normal detrusor pressure is above 30 cm/H_2_O. Before CT, all patients were below normal, and after treatment, 100% of patients were within the normal range (Fig. 4E).

All patients had an increase (*p* < 0.05) in voided volume. The initial mean was 244.00 ± 27.60 mL, and after CT, the mean volume changed to 418.89 ± 32.73 mL (Fig. 4C). The percentage of increase ranged from 4 to 555% (Fig. 4E).

The bladder contractility index (BCI) increased (*p* < 0.05) after MSC transplantation. The initial mean BCI was 44.33 ± 4.85 before CT. After MSC transplantation, the BCI reached 100.56 ± 8.89 (Fig. 4D). According to D'Ancona [2], the desired BCI is above 100. Initially, all patients had a BCI below the desired level. After CT, patients 4, 7 and 9 reached normal values. The other patients, despite not reaching the reference value, showed improvements in BCI that ranged from 64 to 291% (Fig. 4E).

### International Consultation on Incontinence Questionnaire-Short-Form (ICIQ-SF)

**Fig. 5:**
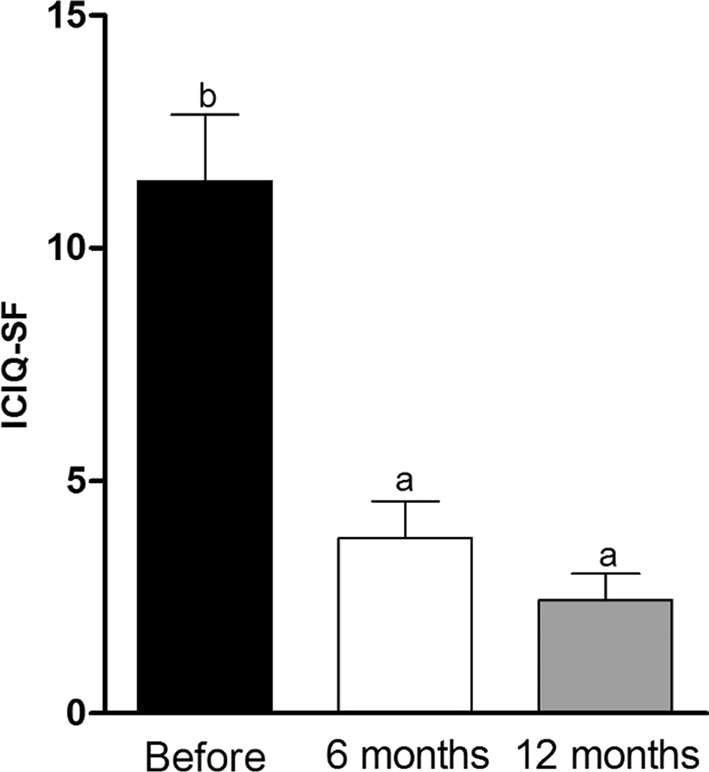
Mean Values ± Standard Error of the Mean of the International Consultation on Incontinence Questionnaire – Short-Form (ICIQ-SF) score obtained in the clinical evaluation before CT, 6 and 12 months after ADSCs transplantation (Statistics: t-Student Peer, p < 0.05)

The International Consultation on Incontinence Questionnaire-Short-Form (ICIQ-SF) scores obtained before and at 6 months and 12 months after ADSCs transplantation showed decreases after treatment (*p* < 0.05). The initial mean ICIQ-SF score before CT was 11.44 ± 1.43. Six months after MSC transplantation, the score increased to 3.77 ± 0.80, and after 12 months, it reached 2.44 ± 0.56 (Fig. 5).

## Discussion

DU is a disease that does not yet have an effective treatment. We demonstrated that CT with ADSCs could be used to treat this disease efficiently, as they were able to increase the maximum flow, the mean flow and volume urinated as well as reduce the residual volume in uroflowmetry exams. Furthermore, treatment with ADSCs improved the efficiency of voiding. CT was still able to increase maximal flow, maximal detrusor pressure, voided volume and BCI in the pressure flow study. This is the first report that demonstrates that ADSCs transplantation can bring these benefits to patients diagnosed with DU.

Only one previous case report and one study examined the use of stem cells to treat underactive bladder. These data were produced by the same research group [8, 13]. The case report describes only one patient, and the stem cells had a muscular origin. The results indicated reduced cystometric capacity, and the patient acquired the ability to urinate small volumes. However, the patient remained dependent on catheterization, and follow-up was carried out up to one year after the transplant. The second study, which used cells derived from autologous muscle, reported an improvement of 83% in the patients studied, although only 69% stopped using intermittent catheterization. Moreover, in both studies, the therapy was considered safe, as there were no adverse effects, including the absence of hematuria, urological emergencies and/or infections [8, 13]. Nonetheless, importantly, despite being produced by the same group of researchers, these studies contain no standardization in the nomenclature of the type of cell that was used. In both studies, the cells were purchased from Cook MyoSite, which describes the cells as human skeletal muscle myoblasts on its website. However, elsewhere, the website also describes the cells as primary undifferentiated human muscle cells, similar to myoblasts (myoblast-like, nondifferentiated primary human muscle cells) from a single donor (Cook, 2022). In view of the above, there is still doubt regarding the level of differentiation of the cells used. Regardless, they are assumed to be cells of mesenchymal origin, but it remains unclear whether they are already committed to the muscle differentiation route.

The present research used adipose tissue as a source for the extraction of mesenchymal stem cells, which is an easily accessible source that has a larger pool of stem cells [17], thereby facilitating the extraction, isolation and production of the desired amount of cells for transplantation. In addition, we used two transplants, 30 days apart, with 2 × 10^6^ cells/transplant. In contrast, Levanovich [8] used 250 × 10^6^ cells for a single transplantation. Gilleran [13] used 125 × 10^6^ cells in the first transplant, and in some cases, a second transplant of 125 × 10^6^ cells was performed 8 months after the first transplant. Our results suggest that the amount of cells used and the time between the two transplants was adequate and should be considered in future studies validating this therapy. We also emphasize that the number of cells used in this study is much smaller than that proposed by Levanovich [8] and Gilleran [13]. We used 62.5× fewer cells than the other authors and obtained superior results. We believe that the superiority of our results is due to the use of mesenchymal stem cells, which are multipotent cells [18, 19]. Levanovich [8] and Gilleran [13] used cells of mesenchymal origin. However, they were already committed to the muscle differentiation route. Therefore, they were unipotent cells [20, 21]. These cells have the ability to produce specific growth factors to produce muscle cells. Satellite cells, which are usually quiescent, between the myofibril-sarcolemma attachments and the basal lamina are stimulated to proliferate and differentiate into myoblasts [22]. In addition, these unipotent cells can still integrate into the tissue by transdifferentiation [20, 23]. These two pathways can lead to tissue regeneration. MSCs, in turn, can undergo regeneration by transdifferentiation; that is, they adhere to the injured tissue and differentiate into the cells of that organ [24]. In this case, the implanted organ was the bladder. This may be because the injured tissue itself produces growth factors and differentiating factors that induce MSCs to transform into the tissue of the organ that needs regeneration [25]. Another important effect that surpasses the capacity of muscle-derived cells (used by other studies) is paracrine action. This occurs when MSCs migrate to the site of injury and produce endogenous repair factors and anti-inflammatory and anti-apoptotic factors that assist in the recovery of the injured organ/tissue. However, they do not integrate into the tissue matrix of the injured organ [25].

Another factor in the comparison of our study with that of Gilleran [13] is the number of patients evaluated. Our study started and ended with nine patients. The study by Gilleran [13] started with twenty patients and ended with nine. In the article, there is no report on the real reason for the patients' withdrawal from participating in the research. This information would be of great importance to validate the results presented. In our study, 100% of the patients remained in the protocol because the results were significant to the patients’ daily life; therefore, they were motivated to continue the treatment. Another fact to consider is that at the end of the study by Gilleran [13], only one patient achieved a voiding efficiency above 80%, with three patients achieving a voiding efficiency between 40 and 60% and five patients with voiding efficiencies below 20%. Our results show superior efficiency, as eight patients had voiding efficiencies greater than 85%, and only one patient had a voiding efficiency between 40 and 60%. These facts reveal that the number of cells was not a preponderant factor in obtaining the results, since we used a smaller amount (62.5 × fewer cells). However, the cell type (as discussed in the previous paragraph), the timing, and the interval between the two transplants can be important differentials as we explore further ways to increase the efficiency of CT under these conditions. Reducing the number of transplanted cells is a crucial factor in reducing treatment costs and should therefore be considered in future clinical applications. Another important fact is the source of the cells. Our results were superior, and we transplanted cells derived from adipose tissue. Therefore, we suggest that the best source is MSCs from adipose tissue, even considering all the differences in experimental design, and that the time between transplants should be 30 days and not the 8 months proposed by Gilleran [13].

We also emphasize that our patients were followed up for 12 months and had no adverse effects or the occurrence of hematuria, urinary urgency and/or infection. Thus, we consider the therapy safe, which is consistent with Levanovich [8]. However, unlike in Levanovich [8], the MCC in our study did not decrease after CT. Our results indicate that this result only occurred in 55.55% of the patients. Thus, we suggest that this parameter may not be the best choice for assessing the effect of therapy on DU. We also emphasize that the increases in maximum flow, mean flow and urination volume, which occurred in 88.89% of patients in the uroflowmetry exam, are important parameters. However, it is even more important to reduce the residual volume, which was achieved in 100% of the patients. We also highlight that 77.78% of the patients had a residual volume < 50 mL after the procedure, which is considered normal [2]. Only two patients maintained a residual volume greater than > 50 mL. However, these two patients showed a significant reduction in residual volume, on the order of 56 and 79%. One of the patients had a residual volume of 1,800 mL, and after therapy, the volume decreased to 800 mL. The other had an initial residual volume of 360 mL that decreased to 80 mL. These values are significant, even though patients did not achieve normal values.

We also suggest that the parameters obtained in the study of flow pressure, maximum flow, urinary volume, maximum detrusor pressure and BCI are important in evaluating the effects of CT. In our patients, all of these values increased in 100, 100, 88.89 and 100% of the patients, respectively, which confirms the therapeutic effect. These data are unprecedented in the literature for this type of study, and Levanovich [8] and Gilleran [13] did not use these parameters in their assessments. However, we consider them essential for understanding the effects of CT on DU.

These quantitative results are supported by the 67.54% reduction in the ICIQ-SF score 6 months after treatment and 78.60% 12 months after treatment. This is an important, condition-specific quality of life questionnaire related to urinary incontinence [26]. The high scores before CT, which were extracted from the patients' reports, showed that they did not have their desired quality of life and that they even limited their routines due to voiding dysfunction. After CT, all patients reported significant improvement in their quality of life and even returned to routine activities that had been interrupted.

All patients maintained voiding diaries and underwent clinical follow-up with laboratory tests. No complications were reported at any time. Moreover, after 12 months of CT, 77.78% of patients no longer used intermittent clean catheterization, and 22.22% reported a reduction from five daily catheterizations to two. Biochemical tests did not indicate any change in renal function (data not shown).

## Conclusions

This novel and pioneering revealed that CT with ADSCs may be a new and safe therapeutic option for the treatment of DU.

## Supplementary Information

**Additional file 1**. Supplementary Material I.

**Additional file 2.** Supplementary Material II.

## Data Availability

All data generated or analyzed during this study are included in this published article.

## Abbreviations

ADSCs: Adipose tissue-derived stem cells
CEP: Human Research Ethics Committee
CeTroGen: Center for Studies in Stem Cells, Cell Therapy and Toxicological Genetics
CONEP: National Research Ethics Committee
CT: Cell therapy
DU: Detrusor underactivity
FICF: Free and Informed Consent Form
HUMAP: Maria Aparecida Pedrossian University Hospital
ICIQ-SF: International Consultation on Incontinence Questionnaire-Short Form
MCC: Maximum cystometric capacity
MSCs: Mesenchymal stem cells
