# Supplementary material for: Autologous adipose-derived mesenchymal stem cell therapy reverses detrusor underactivity: open clinical trial
Source: Stem Cell Res Ther. 2023 Apr 5;14:64. doi: 10.1186/s13287-023-03294-8 (PMC10074857; doi:10.1186/s13287-023-03294-8)
Supplement: Supplementary file 1 — Additional file 1. Supplementary Material I. [file 13287_2023_3294_MOESM1_ESM.docx]

Supplementary Material I

Extraction:

The lipoaspirate was centrifuged for 10 minutes at 2000 rpm. Phosphate buffer solution (PBS) was aspirated, leaving only the adipose tissue. The vials were filled with PBS and centrifuged again, as described above. This process was repeated two more times. A solution of Collagenase type I (Gibco Life Technologies, Brazil, catalog number 17100, lot 2144866) diluted in PBS was added to the adipose tissue. It was incubated for 30 minutes at 37°C with homogenization every 5 minutes. Enzyme activity was neutralized with DMEM (Eagle Dulbecco's Modified Low Glucose Culture Medium - Sigma®, USA, catalog number D5523, lot SLBW2368) containing 10% fetal bovine serum (Gibco Life Technologies, Brazil, catalog number 12657, lot 210480k). New centrifugation was performed for 10 minutes at 2000 rpm. Subsequently, the supernatant was discarded. 10 ml of red blood cell lysis solution was added to the pellet, homogenized, and incubated for 5 minutes. New centrifugation was performed for 10 minutes at 1600 rpm. The supernatant was discarded, and the pellet was resuspended in 3ml of culture medium - DMEM supplemented with 10% fetal bovine serum and seeded in one well of a 6-well culture plate. The culture was carried out in an incubator at 37ºC and 5% CO_2_. After 24 hours, the culture medium was changed.

Cultivation

The mesenchymal stem cell culture was maintained in DMEM low glucose culture medium supplemented with 10% fetal bovine serum at 37°C with 5% CO_2_. The culture medium was changed every 72 hours. Upon reaching 80% confluency, the cells were washed three times with PBS. The cells were collected by enzymatic dissociation (0,5 mL trypsin 0.025% at 37°C). Trypsin was inactivated with 1mL of supplemented DMEM. The cell solution was transferred to a 15mL conical tube and centrifuged for 5 minutes at 1,200 rpm. The supernatant was discarded, and the cells were homogenized in 5 mL supplemented DMEM. The cells were then plated in a 25 cm^2^ bottle. The cultivation was carried out in the same way as described above. When the flask reached 80% confluence, the cells were washed with PBS, enzymatically dissociated, and plated in two 75cm^2^ bottles. The cells were released from the flask with the aid of trypsin, and a 10µL sample of a solution containing the stem cells was placed in a microtube for counting and analysis of cell viability by trypan blue (1:1). This process was repeated until 2.10^6^ mesenchymal stem cells were obtained.

Transplant

When the cells reached 2.10^6^, the cells were washed three times with PBS and enzymatically dissociated. Enzyme activity was inactivated with 2 ml of DMEM culture medium supplemented with 10% fetal bovine serum. Then, the cells were transferred to a 15 mL conical tube. The conical tube was centrifuged for 5 minutes at 1200 rpm. Subsequently, the supernatant was aspirated with the aid of a Pasteur pipette. The cell pellet was resuspended in PBS. Centrifugation was performed for 5 minutes at 1200 rpm. This process was repeated two more times. The pellet was resuspended in 5 ml of sterile saline (NaCl 0,9%) and placed in a 10 ml syringe. The material was sent to the Urology Outpatient Clinic for transplantation.

Differentiation and immunophenotypic

The differentiation was confirmed by the presence of lipid droplets, an extracellular matrix rich in calcium and glycosaminoglics after staining with Oil Red (Sigma®, USA, catalog number O0625, CAS 13200-6-5, lot SLBH0251V), Alizarin Red (Sigma®, USA, catalog number A5533, CAS 130-22-3, lot MKBR6276V) and Alcian Blue (NEON, Brazil, CAS 33864-99-2, lot 21355), respectively (Fig. 1A–D). The immunophenotypic profile of mesenchymal stem cells expressed the CD90 (Pharmingen BD, USA, CAT 561557, lot 7209646) and CD105 (Pharmingen BD, USA, CAT 562408, lot 7222945) markers. It did not express CD133 (Pharmingen BD, USA, CAT 566594, lot 7129908) and CD34 (Pharmingen BD, USA, CAT 560941, lot 7033536). So, this result corroborated that these are mesenchymal stem cells (Fig. 1E), the second protocol of Schweich et al., 2021.


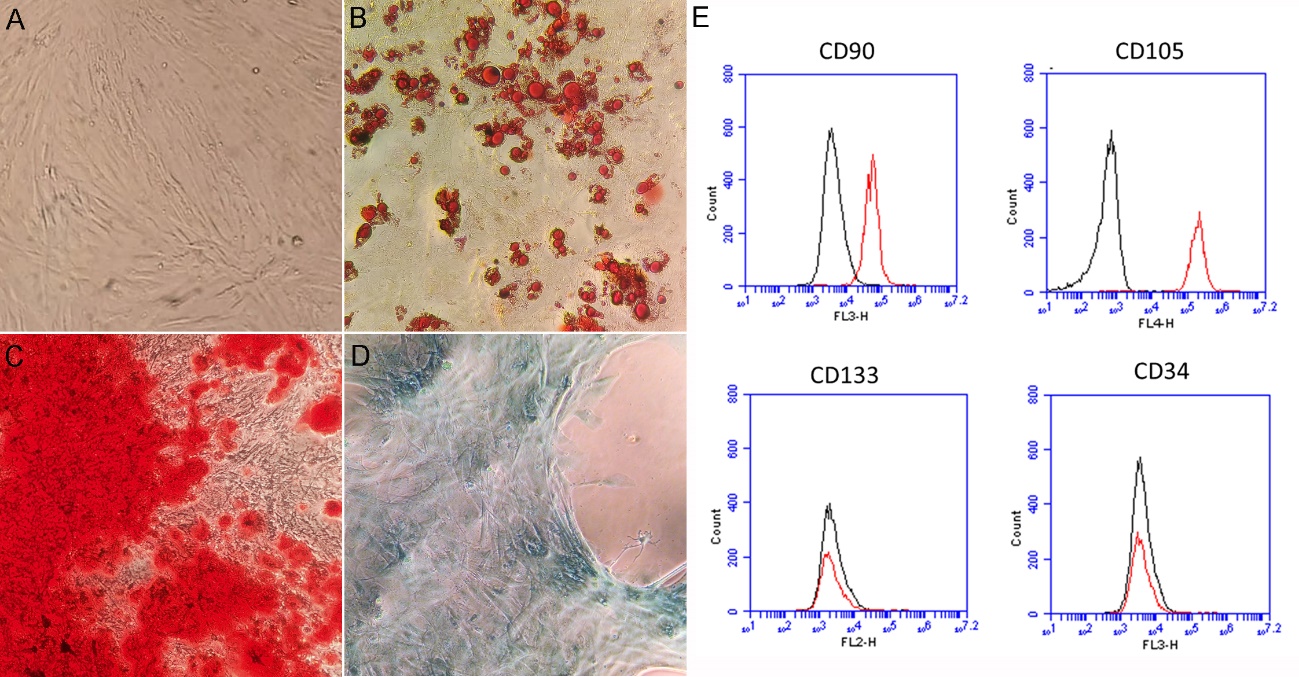


Figure 1S. Morphology, characterization and differentiation potential of adipose-derived stromal cells–(**A**) Undifferentiated culture demonstrating cells with fibroblast characteristics, (**B**) adipogenic differentiation culture and lipid vacuoles stained with Oil Red O, (**C**) osteogenic differentiation culture and calcium deposits stained with Alizarin Red and (**D**) chondrogenic differentiation culture and glycosaminoglycan-rich extracellular matrix stained with Alcian Blue. (**E**) Immunophenotypic profile of mesenchymal stromal cells. Cells expressed the CD 90 and CD105 markers and did not express CD133 and CD34. Image source: own author

Schweich-adami LC, Silva RA, Baranoski A, Kassuya CAL, Antoniolli-Silva ACMB, Oliveira RJ. Effects of Adipose-derived Stem Cells in the Treatment of Knee Osteoarthritis: A Case Report in Brazil’s Unified Health System. Revista Brasileira de Ortopedia. 2021; doi.org/ 10.1055/s-0041-1733797. ISSN 0102-3616.
