# Supplementary material for: Autologous adipose-derived mesenchymal stem cell therapy reverses detrusor underactivity: open clinical trial
Source: Stem Cell Res Ther. 2023 Apr 5;14:64. doi: 10.1186/s13287-023-03294-8 (PMC10074857; doi:10.1186/s13287-023-03294-8)
Supplement: Supplementary file 2 — Additional file 2. Supplementary Material II. [file 13287_2023_3294_MOESM2_ESM.docx]

Supplementary Material II

The individuals underwent the cystoscopy procedure, with intravesical mesenchymal stem cell transplantation, on an outpatient basis. After the procedure, the individuals remained under observation in the procedure room, in the supine position with the head elevated at 45 degrees for 60 minutes under the care of the medical and nursing staff.

Vital signs (blood pressure, heart rate, respiratory rate, axillary temperature and oxygen saturation) were measured by the nursing team during the period of stay at the health unit. Patients received oral hydration and during the period were evaluated for voiding symptoms of pain, bleeding and voiding discomfort.

At the end of this period, the individuals were discharged with guidance regarding possible complications related to the urethrocystoscopy procedure and intravesical mesenchymal stem cell transplantation. Patients underwent a prophylaxis protocol with antibiotic therapy. After discharge, individuals received the drug Levofloxacin 500mg and were instructed to take the drug once a day for 3 consecutive days at the same time. If necessary, they were instructed to use paracetamol 500mg orally every 6 hours for analgesia in case of pain.

Patients returned to the Urology outpatient clinic for medical evaluation every 15 days for the first 2 months and monthly from the second to the sixth month. The patients underwent laboratory tests (complete blood count, urea, creatinine, sodium, potassium, glucose, type 1 urine and urine culture) 15 days after the first and second transplants and every 60 days from the second to the sixth month post-transplant.
